# A multi-stakeholder fuzzy best–worst method analysis of key factors in remanufacturing production processes

**DOI:** 10.1038/s41598-025-31401-7

**Published:** 2026-02-02

**Authors:** Can Miao Gao, Kuan Yew Wong, Muhd Ikmal Isyraf Mohd Maulana

**Affiliations:** Faculty of Mechanical Engineering, Universiti Teknologi Malaysia, Skudai, 81310 Malaysia

**Keywords:** Remanufacturing, Production process, Stakeholders, Multi-Criteria Decision Making, Fuzzy Best-Worst Method, Business and management, Business and management, Engineering, Mathematics and computing

## Abstract

Remanufacturing is a core principle for achieving sustainable manufacturing. However, a comprehensive understanding of remanufacturing production process factors is lacking, as is adequate consideration of stakeholder perspectives and the relative importance of key factors. This study addresses these gaps by examining remanufacturing production processes from a multi-stakeholder viewpoint. It identified the key factors and then evaluated them using the Fuzzy Best-Worst Method (FBWM). The Fuzzy Analytic Hierarchy Process (FAHP) was used for secondary verification. Evaluation results were compared across five stakeholder groups: a scholar, an engineer, an original equipment manufacturer (OEM) manager, an independent remanufacturer (IR) manager, and a consumer. The analysis reveals that “Process Planning” (23.71% average weight) and “Generic Process” (20.24% average weight) are the foundational factors. While specific priorities differ slightly, the engineer and managers (OEM and IR) align on prioritizing these two factors. In contrast, the scholar focuses on innovation within a Specific Process, and the consumer prioritizes factors directly related to the final product. Based on these findings, the study proposes three guiding principles for implementing remanufacturing production processes and explores how stakeholder priorities influence academia, business operations, and the overall remanufacturing market. The main contribution lies in providing critical support for government policy-making and offering reference material for academic research in this area.

**Supplementary Information:**

The online version contains supplementary material available at 10.1038/s41598-025-31401-7.

## Introduction

 The manufacturing industry contributes to the global economy, accounting for 21.4% of global Gross Domestic Product (GDP) in 2022, according to the United Nations Industrial Development Organization^1^. However, this sector also involves substantial resource consumption, utilizing 15% of global energy and 35–40% of materials for industrial production^2^. Consequently, optimizing resource utilization and minimizing waste have become crucial for achieving sustainable development within manufacturing^3^.

Among various sustainable practices, remanufacturing stands out as particularly effective^4^. Its core concepts include enabling used parts to regain functionality and value comparable to that of new products, reducing resource consumption and waste while effectively meeting demand for upgrading obsolete products, and offering economic benefits^4,5^ to maximize the value of finished products.

The remanufacturing production process serves as a critical link between discarded components and remanufactured goods. For example, laser remanufacturing processes enhance raw material utilization rates^6^. The production process of remanufacturing is also closely related to the supply chain^7^, factory production capacity, production sequence, process parameters, and resource allocation^8^. The existing research is highly targeted but lacks a comprehensive perspective and integration of influencing factors.

Some research has begun to explore the association between remanufacturing factors and production processes, including the attributes of influential factors, analyzed potential barrier factors^9^, and examined performance factors^10^. These explorations have remained mainly descriptive, with fewer studies analyzing the relative importance of these factors.

Incorporating multiple perspectives is crucial for the successful implementation of remanufacturing. Stakeholders from different positions may have varying priorities^11^. Therefore, the remanufacturing production process should be the result of multi-stakeholder coordination^12^. However, there is a relative paucity of research documenting stakeholder management in remanufacturing over the past few decades.

Deciphering the relationships between remanufacturing factors and stakeholders can help avoid pitfalls, and identifying key factors and their importance is a critical step in this “decoding” process. However, existing literature suffers from three gaps: (1) a failure to identify the key factors of remanufacturing production processes comprehensively; (2) a lack of assessment of the relative importance of these factors from a multi-stakeholder perspective; and (3) an unclear understanding of stakeholders’ perceptions of production processes factors in remanufacturing production processes.

Considering these gaps, this study aims to explore three key questions: RQ1: What are the key influential factors of remanufacturing production processes? RQ2: What is the relative importance of these factors from a multi-stakeholder perspective? RQ3: What are the differences in the perceived importance of these factors among different stakeholders?

This study addresses existing research gaps by (1) identifying the key influential factors in remanufacturing production processes, (2) evaluating the relative importance of these factors from a multi-stakeholder perspective, and (3) analyzing the differences in perceived importance across various stakeholder groups. The novelty of this work stems from its integration of detailed perspectives. Previous research often lacked comprehensive inclusion of diverse viewpoints; in contrast, this study innovatively incorporates assessments from five distinct stakeholders, allowing for a more in-depth analysis of individual priorities. Furthermore, the research departs from previous macro-level commentary by systematically examining the production process level, thus highlighting the critical role of remanufacturing process flow in achieving sustainable manufacturing.

This study is structured into six sections. Section 2 identifies existing research gaps through a comprehensive literature review. Section 3 outlines the methodology employed in the study. Section 4 details the data collection and analysis processes, including the calculation results of the Multi-Criteria Decision Making (MCDM) method. Section 5 discusses the similarities and differences in stakeholders’ perspectives on key factors in remanufacturing processes and evaluates their implications. Finally, Section 6 provides a summary of the study and discusses future research directions.

## Literature review

This section explores three core areas: multi-stakeholder perspectives, key factors of remanufacturing production processes, and importance of these factors. Literature searches were conducted primarily through academic databases, using keywords including “remanufacturing”, “process”, “production”, “factor”, “importance”, “weight”, “group decision making”, and “stakeholder”. To ensure the relevance of the literature, the screening criteria required that the literature abstract contain at least “remanufacture*” (where “*” is a wildcard) along with another search term. After a two-stage assessment process, including initial screening and in-depth review, the final set of literature for this study was identified from the candidate literature.

### Stakeholder theory

Since its introduction in the 1980s, “stakeholder theory” and “stakeholder thinking” have established a position in practice^13^. Stakeholders’ characteristics are often overlooked^14^. This neglect can leave companies unprepared for sudden competition or result in the misallocation of resources to work processes with little to no substantive demand. Additionally, according to Akano et al.^7^ and Huang et al.^15^, stakeholders under specific remanufacturing models can collaborate, resolve conflicts, and allocate resources to jointly achieve objectives across quality, cost, profit, and brand dimensions.

In the field of remanufacturing, the application of stakeholder theory remains in its early stages. Existing studies have begun to examine the perspectives of different stakeholders, such as labor within the remanufacturing industry^16^; and Original Equipment Manufacturers (OEMs) and Independent Remanufacturers (IRs)^7^; the roles of consumers, businesses, and governments in promoting remanufacturing capabilities^17^; and the balanced remanufacturing decisions among producers, consumers, recyclers, and dismantlers^18^. Table 1 offers an overview of stakeholders in the remanufacturing industry to enhance understanding of this issue.

**Table 1:** Overview of existing research from the perspective of remanufacturing stakeholders.

Year	Reference	Description	Organizations					Individuals									Number of stakeholders
			Governments	Social groups	OEMs	IRs	Suppliers	Scholars	Consumers	Shareholders/ Investors	Managers/ Directors	Supervisors/ Coordinators	Consultants	Engineers	Scavengers/ Collectors	Decomposers/ Disassemblers	
2011	Hatcher et al.^19^	Design for remanufacture			√			√									2
2015	Kafuku et al.^20^	Investment decision issues			√					√							2
2017	Priyono^21^	Understanding the benefits of the product-service system for involved parties in remanufacturing			√	√			√		√	√		√			6
2019	Zhao et al.^22^	Decision for pricing, service, and recycling of closed-loop supply chains considering different remanufacturing roles			√	√	√										3
2019	Sakao and Sundin^23^	Improve remanufacturing			√			√	√								3
2019	Sitcharangsie et al.^24^	Optimize remanufacturing outcomes						√									1
2021	Okorie et al.^25^	A triple bottom-line examination of remanufacturing	√	√	√			√	√								5
2021	Akano et al.^7^	Stakeholder considerations in remanufacturability decision-making			√	√			√								3
2021	Akano et al.^26^	Decision-making factors representing different stakeholders			√	√		√	√								4
2022	Shah and Bookbinder^18^	Balanced development of remanufacturing decisions			√				√						√	√	4
2022	Moroni et al.^17^	Remanufacturing and its impact on stakeholder engagement			√				√		√	√	√	√			6
2022	Feng et al.^27^	Remanufacturing supply chain with government participation considering consumers’ preferences	√		√		√		√								4
2022	Pratapa et al.^28^	Role of standards as an enabler in a digital remanufacturing industry			√		√	√			√	√					5
2023	Huang et al.^15^	Equilibrium strategies and chain members’ profits			√	√	√										3
2023	Wang et al.^29^	Recycling legislation, models and methods	√		√	√			√								4
2023	Fang et al.^30^	The choice of remanufacturing strategy			√	√											2
2023	Belbağ and Belbağ^31^	Consumer behavior in purchasing remanufactured products							√								1
Individual Summaries			3	1	15	7	4	6	10	1	3	3	1	2	1	1	
Aggregate			30					28									
2025	This Study	Differences in perception of remanufacturing process factors among different stakeholders			√	√		√	√					√			5

According to the statistical data in Table 1, organizational stakeholders were considered slightly more often (30 times) than individual stakeholders (28 times). Studies focusing on organizational behavior, particularly concerning OEMs and IRs, which are the most active organizational forms in remanufacturing transactions, are more common and attract the most attention. However, organizational behavior primarily centers on decision outcomes, whereas crucial details such as information, resources, channels, planning, and execution are controlled by individuals with specific job responsibilities. This organizational-oriented research approach risks oversimplifying the problems, underscoring the necessity of gathering input directly from individual stakeholders.

Individual stakeholders comprise nine types of personnel from various sectors, but the distribution of research attention is uneven. Consumers’ perspectives receive the most focus, followed by scholars, while other individual stakeholders receive comparatively less attention. Notably, few studies solicit input directly from remanufacturing engineers. In practice, engineers are pivotal; they are responsible not only for product design, process improvements, and technological upgrades but also play key roles in production efficiency, quality control, and cost optimization. Given their function as a bridge between theory and practice, the expertise of engineers should not be overlooked.

### Key factors that have been investigated in the remanufacturing field

**Fig. 1:**
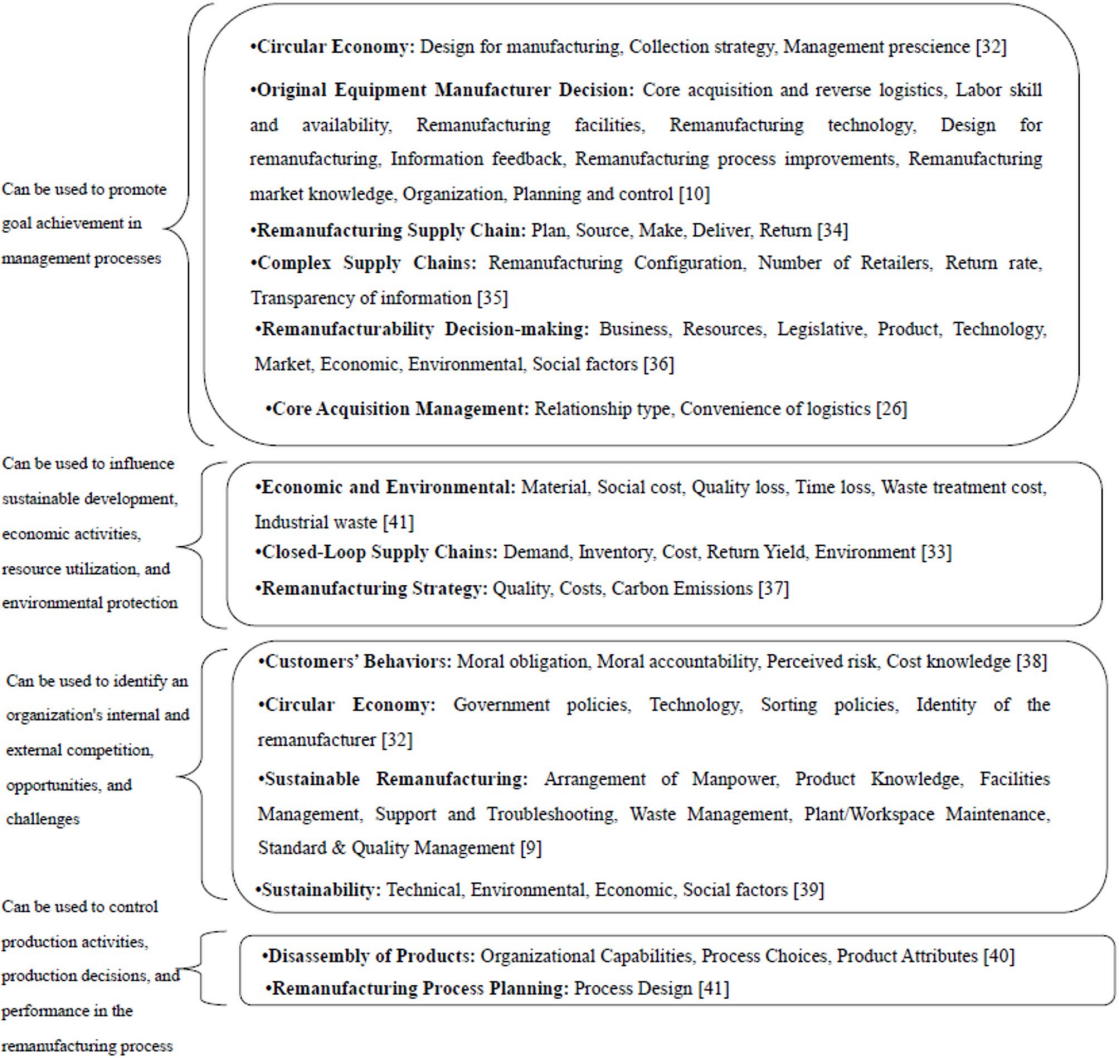
Key factors that have been investigated in the remanufacturing sector.

Scholars have adopted diverse perspectives and methods to investigate the factors that affect remanufacturing. For example, Singhal et al.^32^ and Duberg et al.^10^ focused on factors that can ensure the “used product”is efficient, stable, and controllable during the reshaping process. Ropi et al.^33^ examined the environmental and economic factors in remanufacturing, highlighting their uncertainty. In addition, several factors, either from external or internal sources, indirectly provide a breeding ground for remanufacturing development and change. Figure 1 summarizes some typically investigated influential factors in remanufacturing.

Figure 1 reveals two main trends in research. Most studies place the influencing factors of remanufacturing within a macro framework, such as the circular economy or closed-loop supply chains, treating them as a sub-stage of a more practical problem. While this approach promotes interdisciplinary integration, the definition of key factors remains unclear and unfocused due to the vast and complex nature of the research topic.

Other studies focus on specific technical details, such as the disassembly and reassembly of old products. Although this type of research helps to solve specific engineering problems, its focus is scattered across various stages of the study, lacking systematic summarization and comparison, making it difficult to form a comprehensive understanding. Therefore, research on remanufacturing production processes needs to integrate these two trends more effectively, that is, to consider the role of remanufacturing factors within a larger system while focusing on technical details, to achieve a clearer and more comprehensive understanding. Therefore, studying the "remanufacturing process flow" as an intermediate stage is key to bridging macro-level goals and basic operations.

### Importance of remanufacturing factors

It is important to note that remanufacturing production processes are not limited by fixed factors such as operations, sequences, or equipment. Instead, the importance of these factors often undergoes dynamic changes. As Zemlickienė and Turskis^42^ pointed out, the weight of influential factors in remanufacturing varies with the goals of remanufacturing, and no factor can guarantee the inevitable success of remanufacturing.

Much research on the importance of remanufacturing factors focuses on the influence of these factors. Chaowanapong et al.^43^ extracted the factors affecting decision-making from a practiced process and revealed the association between the ranking of the factors and the outcome of the decision-making. Duberg et al.^10^ categorized remanufacturing factors into two groups based on their importance: necessary factors for the remanufacturing field and supportive factors that enhance the capacity and efficiency of remanufacturing systems, providing a useful theoretical framework for categorizing factor importance.

The calculation or evaluation methods for the importance of remanufacturing factors are also very rich. Subramoniam et al.^44^ demonstrated the relative importance of different factors using a comparison scale. Vasanthakumar et al.^45^ proposed an explanatory structural modeling approach for describing the most dominant and least dominant factors. Singhal et al.^32^ used the Fuzzy Decision-Making Trial and Evaluation Laboratory (FDEMATEL) method to establish causal relationships between factors and determine their importance. In addition, Zhang et al.^46^ established an energy-based mathematical model for each remanufacturing factor. They assessed the performance of these factors based on the differences in input and output. Table 2 summarizes the representative studies on the importance of remanufacturing factors.

**Table 2:** Literature review evaluating influential factors in the field of remanufacturing.

Year	Reference	Focus of the study	Country	Method	Number of factors	Key factors
2013	Subramoniam et al.^44^	Automotive industry	USA/ Europe	Analytic Hierarchy Process	14	The green perception Governmental green initiatives Remanufacturing design Core (or used products) management
2015	Deng et al.^47^	Remanufacturing enterprises	China	Fuzzy Decision-Making Trial and Evaluation Laboratory	15	Policy guidance Social awareness of remanufacturing OEMs information sharing Laws and regulations system Critical remanufacturing technology Remanufacturing design Quality management
2016	Vasanthakumar et al.^45^	Automotive component	India	Interpretive Structural Modeling - Matrix of Cross Impact Multiplications Applied to Classification	20	Management commitment Strategy selection Impact of workplace environment Environmental awareness
2016	D’Adamo and Rosa^48^	Automotive Aerospace Medical equipment	Europe/ China/ USA	Strengths, Weaknesses, Opportunities and Threats Analysis Analytic Hierarchy Process	16	Economic sustainability
2017	Chaowanapong et al.^43^	Automotive part	Thailand	Descriptive statistics	13	Business feasibility Firm’s strategic factors Policy factors
2019	Sitcharangsie et al.^24^	Automotive industry	Thailand	Literature review Classification	18	Core acquisition Component planning and scheduling Disassembly Cleaning
2019	Singhal et al.^49^	E-waste	India	Interpretive Structural Modeling - Matrix of Cross Impact Multiplications Applied to Classification	15	Government incentives Green awareness
2019	Ansari et al.^50^	Supply chain	India	Fuzzy Analytic Hierarchy Process Fuzzy Technique for Order of Preference by Similarity to Ideal Solution	32	Top management support and involvement Mandatory take-back policy Innovation success Price-sensitive consumer
2020	Singhal et al.^32^	Remanufacturing industry	Not Specified	Fuzzy Decision-Making Trial and Evaluation Laboratory	19	Remanufacturing design Management prescience Collection strategy Purchase intention
2020	Duberg et al.^10^	Robotic lawn mower	Sweden	Case study	8	Core acquisition and reverse logistics Labor skills and availability Remanufacturing facilities Remanufacturing process and technology
2020	Liu et al.^51^	Crankshaft remanufacturing	China	Integrated optimization control method	3	Reassembly
2021	Zhang et al.^46^	Machine tool	China	Mathematical model	8	Resource consumption Economic benefits Operating cost
2021	Chen et al.^52^	Used gearbox	Not Specified	Fault Tree Analysis Fuzzy Comprehensive Evaluation	4	Integrated design of process-tolerance
2025	This Study	Remanufacturing process flow	Not Specified	Fuzzy Best-Worst Method	25	Facilities/Equipment Generic process Process planning Testing Specific process Efficiency Product upgrading

Assessing the importance of remanufacturing factors varies in their application and impact. Looking at the focus of the studies in Table 2, many studies concentrated on machinery, equipment, and the automotive industry, which suggests that the extent of the impact of remanufacturing factors on other industries is not clear. It is worth noting that most of the studies (11 out of 13) were conducted in specific country contexts, which might have influenced the results by local socio-cultural factors.

There are differences in the results of “Number of Factors” and “Key Factors” in the table, with the number of factors examined ranging from 3 to 32, and there seems to be no fixed pattern. This reflects the diversity of research methods and perspectives on the factors in the field of remanufacturing. In addition, there was an overlap of key factors across research perspectives, but no two studies had the same key factors. This implies that the importance of remanufacturing factors may be dynamic, changing, and multi-layered.

Then, the strengths of each method should be considered. For example, Interpretive Structural Modeling is suitable for qualitative analysis of factors, while mathematical models are more appropriate for precise quantification and prediction. Table 2 also shows that MCDM is frequently used to evaluate the importance of factors. MCDM can assign specific relevance or weight values to factors, allowing for a more intuitive comparison, assessment, and emphasis on potentially conflicting factors, given the numerous factors in remanufacturing production processes and the involvement of multiple stakeholder perspectives. Fuzzy MCDM is better suited to address the uncertainties commonly present in this context. Table 3 summarizes the existing literature on the application of Fuzzy MCDM in the remanufacturing field.

**Table 3:** Applications of fuzzy MCDM in the remanufacturing Field.

Year	Reference	Objective	Main method	Advantages	Disadvantages
2012	Jihen et al.^53^	Selection process of the best reverse logistics provider	FPROMETHEE	-Flexibility to reflect complex and diverse preferences	-Involves a lot of fuzzy number operations -Difficult to repeat the verification in different situations
2017	Chakraborty et al.^54^	Identify and apply design criteria for products that improve remanufacturability	FAHP	-Clear hierarchy	-Computationally intensive -Exhaustive pairwise comparisons
2019	Ansari et al.^50^	Supply chain practices for implementing remanufacturing	FTOPSIS	-Effective handling of uncertainty -Results are easy to understand	-Results depend on predefined ideal solutions -Final ranking may be affected by different defuzzification methods
2020	Ansari et al.^55^	Risks and impacts of remanufacturing	FCOPRAS	-High comprehensiveness -Provides a utility degree for each factor	-Unable to capture or quantify possible relationships among factors
2022	Ciptomulyono et al.^56^	Solving the sorting problem of incoming materials in the remanufacturing process	FANP	-Applicable to complex non-linear decisions	-Complexity in the network structure -Exhaustive pairwise comparisons
2022	Govindan et al.^57^	Barrier Ranking for Remanufacturing in the Cable and Wire Industry	FDEMATEL	-Able to identify relationships among multiple factors	-Lack of a consistency measure
2024	Polat et al.^58^	Risk assessment in remanufacturing projects	FSWARA	-Fast and easy data acquisition process	-Results depend on initial ranking -Inability to reflect factor interactions
2025	This Study	The Importance of Key Factors in Remanufacturing Processes	FBWM	-Reduces the number of comparisons -Easier to obtain highly consistent results	-Sensitive to the initial identification of the best and worst factors by experts

FPROMETHEE (Fuzzy Preference Ranking Organization Method for Enrichment Evaluations); FAHP (Fuzzy Analytic Hierarchy Process); FTOPSIS (Fuzzy Technique for Order of Preference by Similarity to Ideal Solution); FCOPRAS (Fuzzy Complex Proportional Assessment); FANP (Fuzzy Analytic Network Process); FDEMATEL (Fuzzy Decision-Making Trial and Evaluation Laboratory); FSWARA (Fuzzy Step-Wise Weight Assessment Ratio Analysis); FBWM (Fuzzy Best-Worst Method).

Several key trends and research gaps can be observed. All listed fuzzy methods (such as FAHP, FTOPSIS, and FANP) effectively capture and address the inherent imprecision and subjective ambiguity in expert opinions; however, they differ structurally. FAHP/FANP focus on weight determination within a hierarchical/network structure, while FTOPSIS focuses on ranking options based on ideal distance. In terms of application areas, most studies on remanufacturing remain at the macro level, focusing on topics such as closed-loop supply chains, supplier selection, or environmental impact assessments, and lack in-depth quantitative analysis of the core production processes in remanufacturing. Notably, the near absence of research applying the efficient and consistent FBWM to assess production process factors in remanufacturing is particularly noteworthy. Therefore, this study fills this methodological and application gap by employing FBWM for the first time to assess the relative importance of key factors in remanufacturing production processes, thus providing a more accurate and practically valuable micro-level weight structure.

### Research gaps

The literature analysis reveals three gaps in research on remanufacturing processes: the definition of key influencing factors is unclear, the opinions of some stakeholders have not been fully considered, and the relative importance weights of each factor remain undetermined. These limitations collectively hinder the effective allocation of resources. Crucially, few studies have applied the efficient and consistent FBWM method to assess detailed process factors in remanufacturing. Therefore, this study contributes by using FBWM to assess the differentiated considerations of multiple stakeholders regarding the key factors in remanufacturing processes, thus providing a more precise and inclusive priority value for each factor.

## Methods

This section is structured as follows: the first stage explains the method used to identify the key factors in remanufacturing production processes; the second stage explains the stakeholder interview approach; and the third stage describes the steps of FBWM.

### Factors labeling method

The labeling or coding method is a widely used data analysis technique in qualitative research. The method tags content segments of analytical value by reviewing the primary literature. Researchers can ultimately arrive at meaningful results by repeatedly comparing and clustering the same markers^59^. The advantage of the coding approach is that it focuses on what has been tagged rather than the underlying meaning of the original text, which effectively prevents a researcher’s subjective thoughts from being confused with the source text^60^.

**Fig. 2:**
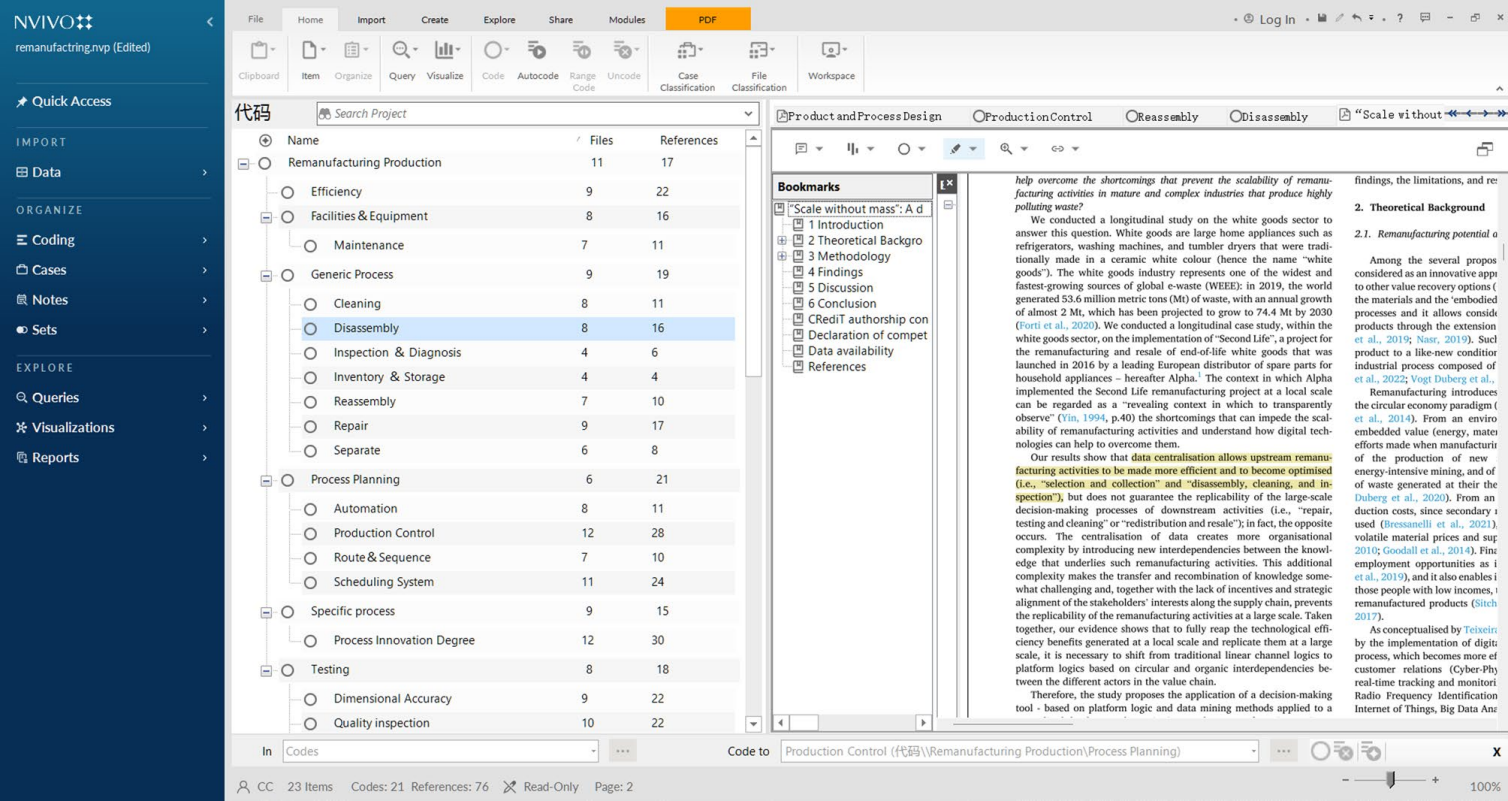
NVivo 12 working interface.

This study applies the factor labeling method, by assigning a unique label to each factor to aggregate potential factors influencing remanufacturing production processes. Among the specialized software available, NVivo 12 was chosen for its comprehensive and flexible features. Figure 2 shows its working interface.

The factor labeling method was adapted from the literature labeling and clustering technique in the N7 + 1 method proposed by O’Neill et al.^61^. The specific steps are as follows:

1. Screening articles: Count the total number of keywords, assess the full-text coverage of keywords in the relevant literature, and retain highly relevant literature.
2. Identify high-frequency words: Conduct word frequency analysis on highly relevant articles to identify potential research hotspots and trends.
3. Open-ended factor markers: Markers are written for the paragraphs or statements in which the high-frequency words are located, with each marker representing a reference point in the factor ensemble.
4. Associative factor labeling: By continuously comparing reference points line by line and paragraph by paragraph, tags are assigned to reference points with identical concepts, forming initial items.
5. Selective labeling stage: Continuously comparing, splitting, moving, reorganizing, clustering items, and renaming categories based on their associations. This process ensures that items of the same category share a common theme, which ultimately forms a list of factors.
6. Generate a list of factors: Check, correct errors (if any), and obtain the result.

### Stakeholder interviews

According to Eisenhardt^62^, selecting multiple stakeholders from various roles as research subjects contributes to the construction of a testable theory. Yin^63^ further emphasized that two essential features are necessary in research: first, the convergence of two or more information sources on the same set of facts or findings; and second, the establishment of a clear logical link between the research questions, the collected data, and the conclusions. These features are critical for ensuring the reliability of the inference process from the research questions to the findings.

The research sample consists of five individuals (a scholar, an engineer, an OEM manager, an IR manager, and a consumer) with practical experience in remanufacturing. The interviewees were selected based on different workplaces, positions, and extensive work experience. Interviews were conducted in various ways, including face-to-face meetings, phone calls, and online platforms. A questionnaire was designed to quantify the key opinions of stakeholders in the form of ratings. Both questionnaires and interviews were conducted using Simplified Chinese. The data were collected from February to June 2025, and all questionnaires were returned.

### Fuzzy best-worst method

To address Research Question 2 (relative importance of factors from multiple stakeholder perspectives), this study selected five representative stakeholders for evaluation. Since these different perspectives inherently involve uncertainty, this paper employs the FBWM to efficiently and robustly handle this issue. The advantages of FBWM in solving this problem are primarily reflected in three aspects: (1) Handling Ambiguity: FBWM incorporates fuzzy theory, effectively addressing the inherent uncertainty and subjectivity in human judgment, thereby more realistically capturing the imprecise attitudes of multiple evaluators. (2) Improving Efficiency and Consistency: FBWM requires only 2n-3 distinct pairwise comparisons ("n" ​​is the number of factors), reducing the cognitive burden on raters and improving data collection efficiency, thereby helping to ensure the consistency and reliability of evaluation results. (3) Precise Weight Calculation: Unlike methods such as FTOPSIS, FBWM does not require pre-setting ideal solutions, making it a better tool for directly calculating and determining the relative weights of factors.

Through FBWM, this study can effectively integrate differentiated evaluations from these five key groups (a scholar, an engineer, an OEM manager, an IR manager, and a consumer). The inclusion of these representative perspectives ensures that the final weighting is not only comprehensive but also has practical guiding value, covering key dimensions of the remanufacturing lifecycle, including theory, technology, organization, and market.

This study employs a refined 0.5–0.9 fuzzy scoring scale to assess the relative importance of factors. A score of 0.5 denotes equivalent importance between compared factors, whereas 0.9 signifies one factor’s substantial superiority over another. Intermediate scores express the subtle weight gradient between factors in increments of 0.1, allowing for more precise quantification of priority distinctions. The FBWM calculation steps used in this paper were developed by Xu et al.^64^ and further applied by Olawore et al.^65^. The calculation procedures of FBWM are as follows:

Step 1: The decision maker needs to determine the decision criteria for the problem. Establish a set of decision criteria C_j_ (j = 1,2, …, n), and identify the best criterion C_b_, and the worst criterion C_w_ among them.

Step 2: Perform fuzzy preference comparisons for the best criterion. The fuzzy preference of the best criterion C_b_ over all the other criteria C_j_ can be determined using a number from 0.5 to 0.9.

The obtained fuzzy best vector would be:

1 $$\:{A}_{b}=({a}_{b1},{a}_{b2},\dots\:,{a}_{bn})$$

where a_bj_ represents the fuzzy preference of the best criterion b relative to the other criterion j. Obviously, a_bb_=0.5.

Step 3: Perform fuzzy preference comparisons for the worst criterion. The fuzzy preference of all the other criteria, C_j_, over the worst criterion, C_w_, can be determined using 0.5 to 0.9. The obtained fuzzy worst vector would be:

2 $$\:{A}_{w}=({a}_{1w},{a}_{2w},\dots\:,{a}_{nw}{)}^{T}$$

where a_jw_ represents the fuzzy preference of the other criterion j relative to the worst criterion w. Obviously, a_ww_=0.5.

Step 4: Determine the optimal fuzzy weights W* = (W*_1_, W*_2_, …, W* _n_). where the optimal fuzzy weight for each criterion ensures consistency in the rater’s fuzzy best and worst comparisons. Specifically, for each pair $\:\frac{{\mathrm{W}}_{\mathrm{b}}}{{{\mathrm{W}}_{\mathrm{b}}\mathrm{+W}}_{\mathrm{j}}}$ and $\:\:\frac{{\mathrm{W}}_{\mathrm{j}}}{{{\mathrm{W}}_{\mathrm{w}}\mathrm{+W}}_{\mathrm{j}}}$, the weights must satisfy the consistency conditions given in Eqs. (3) and (4).

3 $$\:{a}_{\mathrm{bj}}=\frac{{W}_{b}}{{W}_{b}\mathrm{+}{\mathrm{W}}_{j}}$$

and

4 $$\:{a}_{\mathrm{jw}}=\frac{{W}_{j}}{{W}_{w}\mathrm{+}{\mathrm{W}}_{j}}$$

where W_b_, W_j_, and W_w_ are the weights of criteria C_b_, C_j_, and C_w_, respectively.

Step 5: However, Eqs. (3) and (4) do not always hold. To satisfy these conditions for all j, we should find a solution where the maximum absolute differences for all j are minimized. Therefore, we can obtain the constrained optimization problem for determining the optimal fuzzy weights W*, as follows:

5 $$\:s.t.\:\left\{\begin{array}{c}{\sum\:}_{j=1}^{n}{W}_{j}=1,\\\:{W}_{j}\in\:\left[\mathrm{0,1}\right],\:\forall\:j.\end{array}\right.$$

Step 6: To better solve Eq. (5), the model can be transferred to the following constrained optimization problem:

6 $$\begin{aligned} & {\mathrm{min}\:\xi} \\ & {s.t.}\left\{\begin{array}{ll}\left|\frac{{W}{_b}}{{W}{_b}+{W}{_j}}-{a}_{bj}\right|\:\leq\:\xi,\forall\:j,\vphantom{\sum{\int{\int_{\int_{\int_{\int}}}}}} \\ \left|\frac{{W}{_j}}{{W}{_w}+{W}{_j}}-{a}_{jw}\right|\:\leq\:\xi\:,\:\forall\:j,\vphantom{\sum{\int{\int_{\int_{\int_{\int}}}}}}\\{\sum}_{j=1}^{n}{W}{_j}=1,\vphantom{\sum{\int{\int_{\int_{\int}}}}} \\{W}_{j}\in\:\left[\mathrm{0,1}\right],\:\forall\:j.\text{}\end{array}\right.\end{aligned}$$

Step 7: The consistency ratio (CR) is a measure that checks whether the comparison is reliable or not. The larger ξ* is, the higher the consistency ratio is, and the less reliable the comparison is. Based on ξ* and the corresponding consistency index (CI), calculate the CR using the following Eq. 

7 $$\:CR={{\upxi\:}}^{*}/CI$$

The fuzzy scale used in this study is shown in Appendix A (in the Supplementary Information Section below) and the values of the consistency index are presented in Table 4:

**Table 4:** Consistency index (approximate values).

a_bw_	0.5	0.6	0.7	0.8	0.9
CI	0.00	0.20	0.62	1.63	5.23

## Methodology implementation

### Identification of key factors used in remanufacturing production processes

In this study, articles that were of poor quality, had questionable data, or were not compatible with the NVivo 12 software were eliminated.

According to Carpena et al.^66^, keyword clustering leads to an increase in keyword frequency. The keywords mentioned earlier were used as search terms. The articles were then ranked according to reference count and keyword coverage across the full text. Stricter selection criteria were applied: articles with more than 50 keywords and a keyword coverage rate exceeding 0.50% were designated as key articles. Using the factor identification method described in Sect. 3.1, this study identified 25 influential factors, further categorized into 18 items and 7 key factors. The key factors are critical success variables influencing remanufacturing processes, while the items serve as detailed explanations and specific elaborations of these key factors. Table 5 provides an overview of the factors, items, and coding employed in this study.

**Table 5:** Key factors and items list of remanufacturing production processes.

Code	Factors	Items
A	Facilities/Equipment	
A1		Maintenance
B	Generic Process	
B1		Cleaning
B2		Disassembly
B3		Identification & Sorting
B4		Inspection & Diagnosis
B5		Inventory & Storage
B6		Reassembly
B7		Repair
B8		Separate
C	Process Planning	
C1		Production Control
C2		Automation
C3		Route & Sequence
C4		Scheduling System
C5		Standardization
D	Testing	
D1		Dimensional Accuracy
D2		Quality Inspection
D3		Standards
E	Specific Process	
E1		Process Innovation Degree
F	Efficiency	
G	Product Upgrading	

The above list encompasses the core factors of a conventional remanufacturing process, such as “Facilities/Equipment” (A), “Generic Process” (B), and “Process Planning” (C). It emphasizes the importance of ensuring that the product meets the expected standards through “Testing” (D). Notably, the remanufacturing process also has its specificity, which is reflected in both the “Specific Process” (E) and the need for “Product Upgrading” (G). In addition, production “Efficiency” (F) is considered an important indicator of the productivity of the remanufacturing process, while ensuring that the product quality meets the standards.

### Selecting stakeholders

Numerous scholars^3,11,14,17^ have highlighted the method of identifying, classifying, and managing stakeholders, with dichotomous classification being the most common. Some multidimensional schemes have also emerged^67^. In remanufacturing, stakeholders span a normative continuum, such as contractual relationships, economic interests, policy influence, interaction mechanisms, etc. Thus, a dichotomous classification is insufficient for the needs of this study.

This study adopted Miles’^68^ parallel classification model, which operates on the core assumption that stakeholder theory is a management tool, not an ethical mandate. The model acknowledges that organizations build relationships with numerous groups, and it focuses on understanding the processes and outcomes of these interactions for both the organization and its stakeholders. Importantly, this framework does not use absolute, mutually exclusive categories; instead, it recognizes that stakeholder concepts can overlap and diffuse. Accordingly, stakeholders in this paper are grouped into four main categories:

- Influencers: These groups can shape organizational behavior and may either support or hinder the achievement of organizational goals.
- Claimants: These stakeholders present explicit demands, often having established contractual relationships with the organization.
- Collaborators: These parties work with the organization to create shared value. They typically use a passive, indirect strategy to exert influence.
- Recipients: These groups are affected by the organization’s operations but do not directly participate in decision-making or assert claims. Positioned at the end of the supply chain, Recipients may also face risks associated with the company’s activities.

Based on Table 1, OEMs, IRs, consumers, and scholars have been identified as core participants in the field. However, the engineer’s perspective is often overlooked. Due to the above reasons, five representative individual respondents were carefully selected: a scholar with social influence (Influencer); an engineer making explicit demands on the enterprise (Claimant); two managers from different types of remanufacturing firms, both at the management level but representing distinct perspectives, with the OEM manager (Influencer) having direct industry impact, and the IR manager (Collaborator) with indirect impact; and a consumer influenced by demand (Recipient). The background information of the respondents is provided in Table 6.

**Table 6:** Interviewees’ information.

No.	Type	Industry	Role	Position	Educational qualification	Working years
1	Influencer	University	Scholar	Professor	PhD	16
2	Claimant	Remanufacturing	Engineer	Mechanical engineer	Master	12
3	Influencer	Remanufacturing	Manager (OEM)	Manager	Bachelor	11
4	Collaborator	Remanufacturing	Manager (IR)	Supervisor	Bachelor	12
5	Recipient	Construction	Consumer	Purchaser	Bachelor	15

### Application of the fuzzy best-worst method

Each respondent was asked to compare and rate the importance of these seven criteria. They are: “Facilities/Equipment”, “Generic Process”, “Process Planning”, “Testing”, “Specific Process”, “Efficiency”, and “Product Upgrading” (Table 5). After collecting responses, the authors rigorously followed the steps outlined in Sect. 3.3, using FBWM to process the data. The final weight assessment results for each factor are presented in Table 7.

**Table 7:** Optimal weights of the factors and consistency ratios obtained using FBWM calculations.

Factor code	Factors	Weighting						Ranking
		Scholar	Engineer	Manager (OEM)	Manager (IR)	Consumer	Average weight	
A	Facilities/Equipment	0.0559	0.1132	0.1524	0.0800	0.0757	0.0954	6
B	Generic Process	0.1686	0.1994	0.2845	0.1677	0.1919	0.2024	2
C	Process Planning	0.3145	0.3006	0.1897	0.2288	0.1518	0.2371	1
D	Testing	0.1685	0.1421	0.0944	0.1353	0.2563	0.1593	3
E	Specific Process	0.1044	0.0426	0.0759	0.0877	0.0757	0.0773	7
F	Efficiency	0.1043	0.1140	0.1525	0.1354	0.0567	0.1126	5
G	Product Upgrading	0.0839	0.0880	0.0506	0.1650	0.1919	0.1159	4
	CR	0.0510	0.0736	0.0510	0.0771	0.0281		

To ensure the reliability of the research results, Eq. (7) was used to test the logical consistency of the respondents. A prioritization weight matrix can be considered sufficiently consistent when the CR does not exceed 0.1^69^. If the CR exceeds 0.1, pairwise comparisons need to be repeated until the criterion of CR ≤ 0.1 is met. The results of the analysis showed that all the respondents’ best weight ratings of the factors met the consistency ratio requirement.

To enhance the robustness of the findings, the results were validated by comparison with FAHP. The computational steps of Gong et al.^70^ were adopted. First, additional pairwise comparison data for the criteria were collected from the five stakeholders mentioned earlier, and pairwise comparison matrices were generated. The computational steps of FAHP were performed, and the average weights and CRs were calculated (Table 8).

**Table 8:** Optimal weights of the factors and consistency ratios obtained using FAHP calculations.

Factor code	Factors	Weighting						Ranking
		Scholar	Engineer	Manager (OEM)	Manager (IR)	Consumer	Average weight	
A	Facilities/Equipment	0.0699	0.1654	0.0559	0.1075	0.1052	0.1008	5
B	Generic Process	0.1744	0.1567	0.2937	0.1798	0.1529	0.1915	2
C	Process Planning	0.3731	0.2699	0.2267	0.3224	0.1277	0.2640	1
D	Testing	0.1343	0.1588	0.0907	0.1610	0.3950	0.1880	3
E	Specific Process	0.0574	0.0327	0.1052	0.0212	0.0723	0.0578	7
F	Efficiency	0.1067	0.0729	0.0727	0.1006	0.0596	0.0825	6
G	Product Upgrading	0.0842	0.1436	0.1551	0.1075	0.0872	0.1155	4
	CR	0.0712	0.0649	0.0887	0.0931	0.0781		

A comparative analysis of the results from the two MCDM methods shows that, except for the ranking difference between factors A and F (Facilities/Equipment and Efficiency), the rankings of FBWM and FAHP are largely consistent. However, for each respondent, FBWM requires less comparative data and speeds up data collection, resulting in a lower consistency ratio. Therefore, the conclusion favors FBWM, a more efficient and concise MCDM method, which can optimize the weight determination process while maintaining the stability of the results.

## Results and discussion

**Fig. 3:**
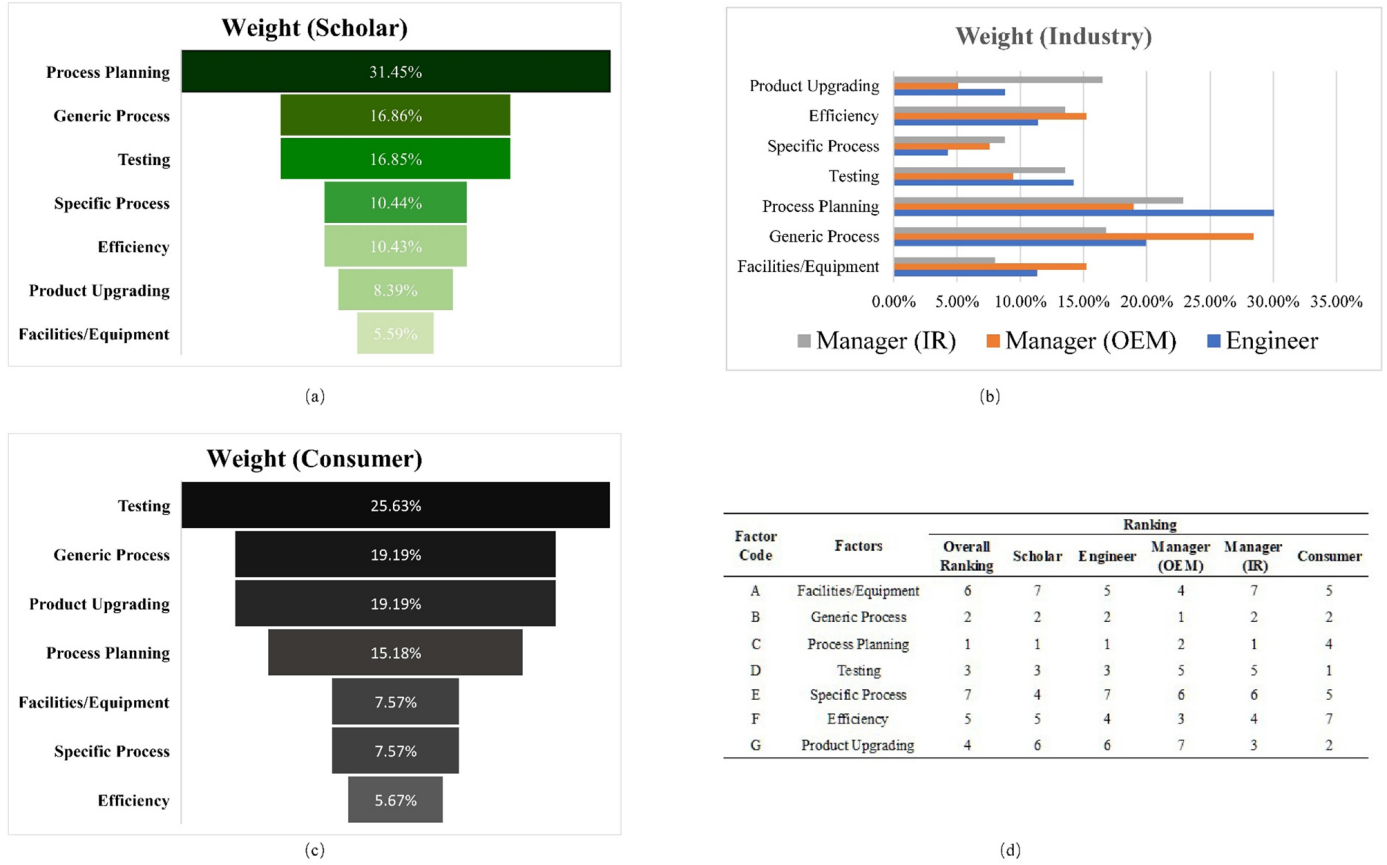
Weights of factors in remanufacturing production processes from the perspectives of stakeholders.

This section presented the evaluation results of five stakeholders on seven key factors in remanufacturing production processes. Charts were used to categorize their focal points and disagreements into three groups for discussion. Based on these findings, a set of practical guidelines for implementing remanufacturing production processes has been developed. Finally, the potential impacts of these perspectives on academia, business operations, and the remanufacturing market were explored. The summary of evaluation results is shown in Fig. 3.

### Scholar perspective

Unlike the profit-driven focus of the commercial perspective, scholars tend to pursue universally applicable theories and models, often contributing innovative ideas for theoretical applications.

As shown in Fig. 3, in the ranking of factors based on the scholar’s perspective, “Process Planning” ranked first with a weight of 31.45%, followed by “Generic Process” with a weight of 16.86%. In comparison, “Facilities/Equipment” was considered less important with the lowest weight of 5.59%. This ranking reflects the unique attitude of the scholar, who believes that the overall planning and organization of remanufacturing production tasks are more critical than the specific preparation of other factors. Notably, “Specific Process” was ranked fourth in the scholar’s ranking with a weight of 10.44%, the highest ranking this factor received in any stakeholder assessment. This phenomenon reflects the high level of academic interest in novel, unique, or context-specific processes.

It is recommended to support scholars in transforming their innovative findings on “specific processes” into industrial application prototypes or small-scale pilot projects. Academic papers should be encouraged to include detailed process implementation roadmaps and potential benefit analyses upon publication. This is expected to alleviate the disconnect between academic innovation and practice, ensuring that scholars’ unique and high focus on “specific processes” translates into core technological competitiveness in the remanufacturing industry.

### Remanufacturing industry perspective (engineer and managers)

The engineer and managers (OEM & IR) as major players in remanufacturing, share common goals and values as well as potential conflicts and contradictions. Through comparison, it was found that the engineer prioritized “Process Planning” (30.06%) and “Generic Process” (19.94%); the OEM manager assigned the highest weights to “Generic Process” (28.45%) and “Process Planning” (18.97%); and the IR manager also placed the most importance on “Process Planning” (22.88%) and “Generic Process” (16.77%). This high level of consensus suggests that “Process Planning” and “Generic Process” are fundamental, foundational factors for achieving an effective remanufacturing production process.

Notably, the OEM manager assigned nearly twice the weight to “Facilities/Equipment” as the IR manager did (15.24% vs. 8.00%). The highest weight for “Product Upgrading” among all the stakeholders was given by the IR manager (16.50%). These factors represent the competitive advantages of OEMs and IRs, respectively. In contrast, none of the three roles prioritized the “Specific Process” factor, indicating a low enthusiasm for innovation within remanufacturing processes.

Leveraging the industry participants’ consensus on “Process Planning” and “Generic Process”, it is recommended to establish a cross-organizational (OEMs and IRs) standard and benchmark platform for common remanufacturing processes. Alternatively, industry associations or third-party organizations can lead the development of best practice specifications for the core “Generic Process”. Simultaneously, recognizing the high importance OEM managers place on “Facilities/Equipment”, promoting equipment sharing or leasing mechanisms can help IR managers acquire higher-quality facility resources without upfront capital investment, thereby narrowing resource gaps in facilities/equipment. This will solidify the industry’s fundamental advantages and promote fair competition.

### Consumer perspective

Consumers play a crucial role in remanufacturing production processes. The results of the consumer assessment, shown in Fig. 3, reflect a user-oriented mindset: the consumer used a product primarily for needs rather than for manufacturing purposes, and therefore showed a high level of concern for “Testing” (25.63%). This factor is important to ensure that the attributes, performance, form, and value of the remanufactured product meet or exceed expectations^71^. The consumer gave the highest weight to “Product Upgrading” (19.19%) among all the stakeholders, which highlights his strong expectations for performance improvement of remanufactured products. In contrast, the consumer showed low interest in topics related to detailed remanufacturing production processes, such as “Specific Process” (7.57%), “Facilities/Equipment” (7.57%), and “Efficiency” (5.67%). This indicates that the core demand of consumers for remanufactured products is “centered around their use value”.

Based on the above results, it is recommended that a remanufactured product testing and quality certification system be adopted and oriented towards “consumer use value”. This system should go beyond existing basic performance testing, setting standards specifically for attributes of high consumer concern (such as durability, functional improvements, and failure rates during the warranty period). Simultaneously, detailed testing and certification labels should be mandated on remanufactured products, clearly explaining how the products meet or exceed expectations. This will help eliminate consumer concerns about the quality and performance of remanufactured products, directly addressing the high focus on " Testing”, thereby increasing consumer willingness to purchase.

### An integrated perspective

The ranking of each factor from each stakeholder’s viewpoint (Fig. 3) highlights cognitive differences regarding the importance of key factors. The results indicate that the “Process Planning” (C) factor took the lead in the overall ranking and most group assessments, highlighting its central role in the overall assessment framework. It was followed by the factors “Generic Process” (B) and “Testing” (D), which also showed high overall importance.

It is worth noting that the rankings of “Efficiency” (F) and “Product Upgrading” (G) fluctuate across groups, reflecting an opposition between short-term interests and long-term development strategies. “Facilities/Equipment” (A) and “Specific Process” (E) are located at the bottom of the overall ranking, which may be because current remanufacturing processes are still in the early stages, resulting in relatively low demand for high-level standards in specific environments.

By comparing and summarizing the perspectives obtained, this study proposes three implementation guidelines for remanufacturing production processes:

1. Building a solid foundation system. This phase focuses on core factors such as “Process Planning” (C), “Generic Process” (B), and “Testing” (D), ensuring the stability and reliability of all production activities in remanufacturing companies through effective planning, organization, command, coordination, and control.
2. Balancing between short-term efficiency and long-term development. Specifically, it is necessary to find an appropriate balance between “Efficiency” (F), which seeks short-term profits, and “Product Upgrading” (G), which promotes long-term development. For example, the integration of efficiency improvement and technological upgrading through technological innovation.
3. Promoting continuous improvement. This deals with the overall optimization of remanufacturing production efficiency, emphasizing the coordinated use of resources such as “Facilities/Equipment” (A), processes, and labor skills. Concurrently, it aims to enhance remanufacturing production technology holistically by advancing the application and refinement of “Specific Process” (E), for specialized products.

### Implications

This section explores the impact of stakeholders’ insights into the key factors in remanufacturing production processes on academia, business operations, and the remanufacturing market.

#### Impact on academia

The scholar’s evaluation of the influential factors of remanufacturing production processes will have multifaceted impacts on academia. Rather than focusing on specific problem-solving, the scholar plans the work with an eye to the interests of the whole. The scholar has emphasized the focus on the “Specific Process” factor, which provides the possibility of opening new research directions. If it stimulates more in-depth discussions, it will promote the systematic development of the remanufacturing discipline.

In addition, the scholar’s assessment of the key factors yields authoritative results that can serve as a crucial theoretical basis for policy formulation, educational content reform, and the establishment of industry standards. Integrating academic research with practice deepens the practicality of the remanufacturing field. This two-way promotion mechanism is of great significance in promoting the overall progress of remanufacturing technology.

#### Impact on business operations

The assessment results from the engineer and managers (OEM & IR) provide perspectives on business operations. At the management level, businesses should prioritize the key factors in remanufacturing production processes, such as “Process Planning” and “Generic Process”, which have a substantial influence on resource allocation under uncertain conditions.

The engineer’s prioritization of the factors reflects the frontline staff’s actual expectations for remanufacturing processes, providing valuable bottom-up feedback. Additionally, OEMs and IRs can compare each other to understand the differences in their decision-making processes for remanufacturing production. This comparative analysis promotes stakeholder collaboration, establishes long-term partnerships, enables risk-sharing, and builds mutual trust.

#### Impact on the remanufacturing market

Conventional views often treat consumer preferences as marketing issues, overlooking their potential connection to remanufacturing processes. Some consumers view production processes as a key indicator of product quality and are even willing to pay a premium for products made with certain processes. This perspective aligns with consumer preferences for “Testing” and “Generic Process” factors as observed in this study. These preferences may manifest in the pursuit of environmentally friendly processes, trust in high-tech processes, or other aspects.

## Conclusions

To address the sustainable challenges brought about by the expansion of the manufacturing industry, traditional manufacturers are exploring remanufacturing strategies. Remanufacturing consists of multiple work stages. Although factors such as cleaning, disassembly, and reassembly have received much attention in generalized remanufacturing processes, few studies have comprehensively considered the key factors of remanufacturing production processes. In addition, early studies have been largely limited to the perspectives of remanufacturers and consumers, overlooking the viewpoints of other stakeholders.

This study has addressed the research questions in the following steps: First, through a systematic literature review and factor labeling, a set of key influencing factors of remanufacturing processes was identified and determined (solved RQ1). Then, several representative stakeholders were selected as evaluators, and fuzzy pairwise comparison data were collected using FBWM to calculate the relative weights of the factors from each stakeholder’s perspective (solved RQ2). Finally, by comparing and analyzing the weight results derived from different groups, the differences in their perceptions of factor importance were quantified and explained (solved RQ3). The differences in the focus of various stakeholders will have a direct impact on academia, business operations, and the remanufacturing market.

This study serves as a pioneering work with a broadly applicable methodology that can be generalized to various stakeholders and enterprises. The results of this study not only fill a gap in existing literature but also provide practical guidance for remanufacturing practices, as well as new perspectives for making remanufacturing decisions, improving efficiency, and promoting sustainable development. Traditional manufacturing companies can focus on the seven key factors of remanufacturing production processes identified in this study to enhance and accelerate the adaptation of their remanufacturing activities.

The authors suggest that this research could be expanded in the following ways: increasing the number of stakeholders or interviewees, utilizing the key factors to develop more reasonable remanufacturing standards, and applying remanufacturing process guidelines to improve productivity. In addition, while this study focused on existing product technologies, future research could consider the evolution of product technologies, such as remanufacturing production processes for special materials. These directions will provide innovative ideas and approaches to address the challenges in remanufacturing.

## Supplementary Information

Below is the link to the electronic supplementary material.

Supplementary Material 1

## Data Availability

The datasets used and/or analyzed in this study are available from the first author on reasonable request.
