# Correction: A multi-stakeholder fuzzy best–worst method analysis of key factors in remanufacturing production processes

**DOI:** 10.1038/s41598-026-41416-3

**Published:** 2026-03-11

**Authors:** Can Miao Gao, Kuan Yew Wong, Muhd Ikmal Isyraf Mohd Maulana

**Affiliations:** Faculty of Mechanical Engineering, Universiti Teknologi Malaysia, Skudai, 81310 Malaysia

Correction to: *Scientific Reports* 10.1038/s41598-025-31401-7, published online 02 February 2026

The original version of this Article contained errors in Equation 6.

6 $$\:\mathop{\mathrm{min}\:\xi}\limits_{s.t.}\left\{\begin{array}{c}\left|\frac{{W}{b}}{{W}{b}+{W}{j}}-{a}{bj}\right|\:\leq\:\xi\:,\:\forall\:j,\\\:\left|\frac{{W}{j}}{{W}{w}+{W}{j}}-{a}{jw}\right|\:\leq\:\xi\:,\:\forall\:j,\\\:{\sum\:}{j=1}^{n}{W}{j}=1,\text{}\\\:{W}_{j}\in\:\left[\mathrm{0,1}\right],\:\forall\:j.\text{}\end{array}\right.$$

now reads:

6 $$\:\mathop{\mathrm{min}\:\xi}\limits_{s.t.}\left\{\begin{array}{l}\left|\frac{{W}_{b}}{{W}_{b}+{W}_{j}}-{a}_{bj}\right|\:\leq\:\xi,\:\forall j,\\\:\left|\frac{{W}_{j}}{{W}_{w}+{W}_{j}}-{a}_{jw}\right|\:\leq\:\xi,\:\forall j,\\\:{\sum}_{j=1}^{n}\:{W}_{j}=1,\text{}\\\:{W}_{j}\in\:\left[\mathrm{0,1}\right],\:\forall j.\text{}\end{array}\right.$$

The original Article has been corrected.

