# Supplementary material for: A multi-stakeholder fuzzy best–worst method analysis of key factors in remanufacturing production processes
Source: Sci Rep. 2026 Feb 2;16:4725. doi: 10.1038/s41598-025-31401-7 (PMC12867993; doi:10.1038/s41598-025-31401-7)
Supplement: Supplementary file 1 — Supplementary Material 1 [file 41598_2025_31401_MOESM1_ESM.docx]

Appendix A Fuzzy scoring scale

| **Fuzzy Scales** | 0.5 | 0.6 | 0.7 | 0.8 | 0.9 |
| --- | --- | --- | --- | --- | --- |
| **Linguistic Terms** | Equal Importance | Weak  Importance | Moderate Importance | Strong  Importance | Extreme  Importance |
